# Draft Genome Sequence of Saccharospirillum sp. Strain MSK14-1, Isolated from Surface Seawater Collected at Aburatsubo Inlet in Japan

**DOI:** 10.1128/genomeA.00469-18

**Published:** 2018-05-31

**Authors:** Masumi Hasegawa, Yu Nakajima, Shu-Kuan Wong, Keiji Nakamura, Yoshitoshi Ogura, Tetsuya Hayashi, Kazuhiro Kogure, Susumu Yoshizawa

**Affiliations:** a Atmosphere and Ocean Research Institute, University of Tokyo, Chiba, Japan; b Department of Natural Environmental Studies, Graduate School of Frontier Sciences, University of Tokyo, Chiba, Japan; c Department of Bacteriology, Faculty of Medical Sciences, Kyushu University, Fukuoka, Japan

## Abstract

Here, we report the draft genome sequence of Saccharospirillum sp. strain MSK14-1, isolated from surface seawater collected at Aburatsubo Inlet in Japan. The genome sequence of strain MSK14-1 should contribute to our understanding of the characteristics of the genus Saccharospirillum.

## GENOME ANNOUNCEMENT

Saccharospirillum sp. strain MSK14-1 was isolated from surface seawater collected near the pier of Misaki Marine Biological Station, University of Tokyo (35°09.5ʹN, 139°36.5ʹE; depth, 20 cm), at Aburatsubo Inlet, Misaki, Kanagawa Prefecture, Japan. To isolate this strain, aliquots of 100 µL of seawater sample were inoculated onto a 1/10-strength ZoBell agar plate. After incubation, colonies were picked and reisolated onto a 1/2-strength ZoBell agar plate. The genomic DNA sample was extracted using phenol-chloroform and ethanol precipitation (1). The KAPA HyperPlus kit (for Illumina) (Kapa Biosystems) was used for library preparation, and paired-end sequences (300 bp of each end) were obtained on the MiSeq instrument with MiSeq reagent kit version 3 (Illumina). MiSeq reads were assembled using Platanus version 1.2.4 (2). The genome was annotated using the Prokaryotic Genome Annotation Pipeline (PGAP) at NCBI (3) and reviewed with the Rapid Annotations using Subsystems Technology (RAST) version 2.0 (http://rast.nmpdr.org). The 16S rRNA gene sequence taxonomic identification using EzTaxon (4) confirmed that strain MSK14-1 is most closely related to Saccharospirillum correiae CECT 9131^T^ (97.58% identity). Cells of the genus Saccharospirillum are obligately aerobic, microaerophilic, or facultatively anaerobic heterotrophs. Motility and presence of flagella are species dependent (5). The genome sequence of Saccharospirillum sp. strain MSK14-1 consists of 39 scaffolds (3,751,839 bp in total; *N*_50_, 1,025,043 bp) with median read coverages of 67.0×. The estimated genome size (3.75 Mbp) of strain MSK14-1 was smaller than that of the draft genome sequence of Saccharospirillum impatiens CECT 5721^T^ (4.74 Mbp) available in GenBank. The G+C content (55.4%) of strain MSK14-1 was the same as that of strain CECT 5721^T^. For strain MSK14-1, the PGAP identified 3,550 genes, including 3,487 protein-coding sequences (CDSs), 46 tRNAs, 3 noncoding RNA genes, and 35 pseudogenes. The RAST annotation identified 3,471 CDSs.

The results of the genome annotation showed that strain MSK14-1 has more iron acquisition (such as siderophores) and metabolism-related genes than strain CECT 5721^T^, which was isolated from the aerobic zone of a hypersaline lake (6). Siderophores are high-affinity iron-chelating agents produced by bacteria and fungi growing under low-iron stress. The role of siderophores is to acquire ferric ion as a nutrient (7). In addition, iron chelator utilization protein genes in the category of membrane transport genes were also found from strain MSK14-1, but not from strain CECT 5721^T^. These results indicated that iron metabolism is different between strains MSK14-1 and CECT 5721^T^. Increasing the available genome information regarding Saccharospirillum strains from various environments will provide a better understanding of their lifestyle and evolution.

### Accession number(s).

This whole-genome shotgun project of Saccharospirillum sp. strain MSK14-1 has been deposited at DDBJ/ENA/GenBank under the accession number MIES00000000. The version described in this paper is version MIES01000000.

## References

[1] Marmur J 1961 A procedure for the isolation of deoxyribonucleic acid from micro-organisms. J Mol Biol 3:208–218. doi:10.1016/S0022-2836(61)80047-8.

[2] Kajitani R, Toshimoto K, Noguchi H, Toyoda A, Ogura Y, Okuno M, Yabana M, Harada M, Nagayasu E, Maruyama H, Kohara Y, Fujiyama A, Hayashi T, Itoh T 2014 Efficient de novo assembly of highly heterozygous genomes from whole-genome shotgun short reads. Genome Res 24:1384–1395. doi:10.1101/gr.170720.113. 24755901 PMC4120091

[3] Tatusova T, DiCuccio M, Badretdin A, Chetvernin V, Ciufo S, Li W 2013 Prokaryotic genome annotation pipeline. *In* The NCBI handbook, 2nd ed National Center for Biotechnology Information, Bethesda, MD.

[4] Kim O-S, Cho Y-J, Lee K, Yoon S-H, Kim M, Na H, Park S-C, Jeon YS, Lee J-H, Yi H, Won S, Chun J 2012 Introducing EzTaxon-e: a prokaryotic 16S rRNA gene sequence database with phylotypes that represent uncultured species. Int J Syst Evol Microbiol 62:716–721. doi:10.1099/ijs.0.038075-0. 22140171

[5] Choi A, Oh H-M, Cho J-C 2011 *Saccharospirillum aestuarii* sp. nov., isolated from tidal flat sediment, and an emended description the genus *Saccharospirillum*. Int J Syst Evol Microbiol 61:487–492. doi:10.1099/ijs.0.022996-0. 20363931

[6] Labrenz M, Lawson PA, Tindall BJ, Collins MD, Hirsch P 2003 *Saccharospirillum impatiens* gen. nov., sp. nov., a novel *γ-Proteobacterium* isolated from hypersaline Ekho Lake (East Antarctica). Int J Syst Evol Microbiol 53:653–660. doi:10.1099/ijs.0.02406-0. 12807182

[7] Neilands JB 1995 Siderophores: structure and function of microbial iron transport compounds. J Biol Chem 270:26723–26726. doi:10.1074/jbc.270.45.26723. 7592901

